# Knowledge, attitudes, and practices regarding vector-borne diseases in central Mexico

**DOI:** 10.1186/s13002-021-00471-y

**Published:** 2021-07-21

**Authors:** Joel E. Nava-Doctor, César A. Sandoval-Ruiz, Antonio Fernández-Crispín

**Affiliations:** 1 Laboratorio de Artropodología y Salud. Facultad de Ciencias Biológicas, Benemérita Universidad Autónoma de Puebla, Blvd. Valsequillo y Av. San Claudio. Edificio BIO I, Ciudad Universitaria, Col. Jardines de San Manuel, C. P. 72570 Puebla, México; 2 Laboratorio de Cultura y Educación Ambiental, Facultad de Ciencias Biológicas, Benemérita Universidad Autónoma de Puebla, Blvd. Valsequillo y Av. San Claudio. Edificio BIO I, Ciudad Universitaria, Col. Jardines de San Manuel, C. P. 72570 Puebla, México

**Keywords:** Social representation, Insects, Vector-borne diseases, Mexico

## Abstract

**Background:**

While vector-borne diseases (VBDs) pose an important public health problem worldwide, there is a limited and conflicting knowledge about such illnesses in rural or urban settings. The present study aimed to explore the social representations (SRs) held by people in the state of Puebla, Mexico on insects and the diseases they transmit. Understood as the group of ideas held and shared by a group of human beings which enable them to understand and interpret the world, SRs constitute what could be called a collective science or knowledge of everyday life.

**Methods:**

The present study was conducted in six municipalities in the state of Puebla, wherein an open-ended questionnaire was applied with three age ranges. A total of 360 questionnaires were applied with people dedicated to a variety of activities. The survey data was analyzed to identify the SR’s structure (the central nucleus of the SR and its peripheral system) and the level of organization in order to explore the degree to which the ideas that constitute it are shared and based on consensus. To describe the structure of the SR, a network analysis was conducted and complemented by a correspondence analysis, which also enables the differences between social groups to be identified.

**Results:**

Popular knowledge on insects and VBDs is often limited, even in communities in which more than one-vector insect is found. The elements that were most frequently mentioned in the data, as pertaining to the insect–disease relationship, were mosquitoes and dengue fever, with scorpions (which are arachnids and not insects) receiving the second-highest number of mentions, while other insects such as kissing bugs, flies, and cockroaches were also mentioned as transmitting VBDs. While television was the main information source on VBDs for the residents of these communities, biology books were also mentioned. Chemical control measures (insect repellents) were the most used prevention method, and traditional medicine was the remedy most commonly used to treat insect bites and transmitted diseases. Entomophobia was the main cause for the respondents’ fear and rejection of insects. Beyond the deleterious effects of many insects, those surveyed also recognized a positive relationship with insects due to economic and nutritional benefits they provide.

**Conclusions:**

The present study provides relevant information on how insects and the diseases they transmit are perceived by rural and urban communities. Although the population is aware of dengue fever and the role of mosquitoes in transmitting it, information campaigns are required for other historically neglected VBDs, such as leishmaniasis, Chagas disease, and, even, rickettsiosis. As it is important to understand the impact that these illnesses have on communities further research is required to ensure that better information and guidance is provided on VBDs in order to develop a culture of illness prevention in not only the rural but also the metropolitan communities of the state of Puebla.

**Supplementary Information:**

The online version contains supplementary material available at 10.1186/s13002-021-00471-y.

## Background

Vector-borne diseases (VBDs) are illnesses caused by the bites of various arthropods, such as mosquitoes, ticks, or true bugs [1], and are one of the most significant public health problems worldwide [2].

While the most significant VBDs in the American continent are dengue fever, malaria, leishmaniasis, and Chagas disease, some of them are classified as neglected diseases, given the low levels of knowledge on them and a popular perception that they are of low risk [3–5]. This lack of knowledge and prevention measures in rural communities aggravates the issue [4, 6].

The risk of transmission of one or more of these diseases is high in Mexico due to its geographic and climatic characteristics, allied to its demographic and socioeconomic conditions [7]. Diseases such as yellow fever, dengue fever, zika, chikungunya, malaria, American trypanosomiasis, Lyme disease, and leishmaniasis, among others, develop under favorable ecological and social conditions in which vectors, causal disease agents, susceptible hosts, and reservoirs maintain a constant dynamic of the transmission [8].

Establishing exactly what people know about insects and VBDs is of utmost importance, as is identifying their erroneous beliefs, doubts, and the reasons that hinder collective and individual action, in order that actions can be taken to impede the reproduction of vectors and avoid the use of polluting insecticides or insects repellents. This process would be useful for identifying key factors pertaining to popular knowledge on and the prevention of these illnesses and their main vectors of transmission. For example, while there is little knowledge about the West Nile virus in the neighborhoods of New York, USA, the perception of risk is relatively high and has led to the application of preventive measures and the identification of breeding sites in home gardens (mainly pots, buckets, and bird fountains). Additionally, *Aedes japonicus, Culex pipiens,* and *Culex restuans* were identified as potential vectors of both this virus and other arboviruses that affect human and animal health [9].

Another study that interviewed high school students in French Guiana identified three classifications of individual protection levels (low, moderate, and high) against the chikungunya virus, finding that only 28% the majority of whom were female had developed high-level prevention against this illness [10]. Research conducted in Ethiopia found a low level of knowledge about the prevention of and protection against visceral leishmaniasis. However, this level improved during the 2-year study after the rollout of prevention campaigns which aimed to develop and expand a culture of prevention and presented testimonials from patients, who served as health agents [11]. Fever was detected as the main symptom of malaria by a study conducted in Panama, where people resort to cultural, physical, and medical control measures to avoid the spread of diseases, and the health sector is the main source of public information [12].

Studies on knowledge and risk perception of dengue fever carried out in two municipalities of the state of Morelos in Mexico revealed that households in both communities presented high levels of vulnerability to the disease. One such area of vulnerability was the greater risk to which members of the community are exposed and their inability to either address this risk or recover from the disease itself [13]. The implementation of an educational strategy for the prevention of dengue fever in the southeast of Mexico, in the states of Chiapas and Yucatan, was found to increase children’s knowledge about self-care and, thus, generate a change in the attitude toward the disease in their homes [14]. However, the study found limited knowledge in its subjects about vector insects, given that, despite having also been asked about Chagas disease and its vector of transmission, they identified the mosquito as the main vector in the region and, therefore, dengue disease. Health centers are the main information source for people and most frequently use chemical and physical control measures as prevention methods [15]. While all of the abovementioned papers provide important information on what the authors consider as either correct or incorrect knowledge on and attitudes about VBDs, in general, they assumed that knowledge and attitudes can be “correct” simply by providing better quality information. The theory of social representations enables us to approach the construction of knowledge as a social process that occurs within a group of people. Under this theory, new information is subject not only to the negotiation of meanings, conflicts, contradictions, and resistance, but also factors that may help the acceptance of changes that would enable the community to prevent VBD transmission [16–18].

Social representation theory was originally proposed by Moscovici (1961) to explain how knowledge produced in academic circles is reappropriated by the public. Social representations are understood as the group of ideas held by a human community that allows it to understand and interpret the world, constituting a logical language and creating what could be called a collective science or a knowledge of everyday life [19]. Social representations enable people to understand and control their context, thus rendering it predictable, and to find a certain consistency and stability in the face of the information saturation with which they are faced daily, making the strange familiar by interacting with others. Organized, shared by the same social group, produced collectively, and are socially useful, social representations (SRs) serve as a guide for social practices and constitute structured/social systems of expectations that enable behavioral adjustment or the justification of one’s own or others’ behavior [20]. Based on their systematic meta-theoretical review of the literature on the theory of social representations, De Rosa et al. [18] consider that the theory has been widely used, especially in Europe and Latin America, to address a wide range of issues in such fields as healthcare, environment, education, constructions of identity, and human rights in a truly supra-disciplinary manner.

Social representations are dynamic and often undergo a continuous renegotiation of meanings, although some may be very resistant to change, especially if they are strongly anchored in practices, institutions, or beliefs deeply rooted in social groups. On some occasions, SRs may differ from group to group, thus generating controversy [16]. The structuralist approach enables an understanding of the stable and dynamic elements of an SR [21]. The stable elements constitute its central nucleus and provide the members of a group with a framework of notions capable of generating consensus and integrating individual differences, while the peripheral system is more dynamic, with more concrete and contextualized elements that give meaning to the more abstract and symbolic central elements [22]. The peripheral system incorporates the new ideas provided by the individuals comprising the group and can explain new or strange situations that cannot be explained by the elements of the central nucleus.

Given the suitable environmental and social conditions for the development and spread of VBDs in central Mexico, the present study analyzed the social representations, attitudes, and practices to ascertain the best known insects and VBDs, how knowledge on VBDs is acquired and shared, and the prevention methods and remedies applied.

## Methods

### Study area

**Fig. 1:**
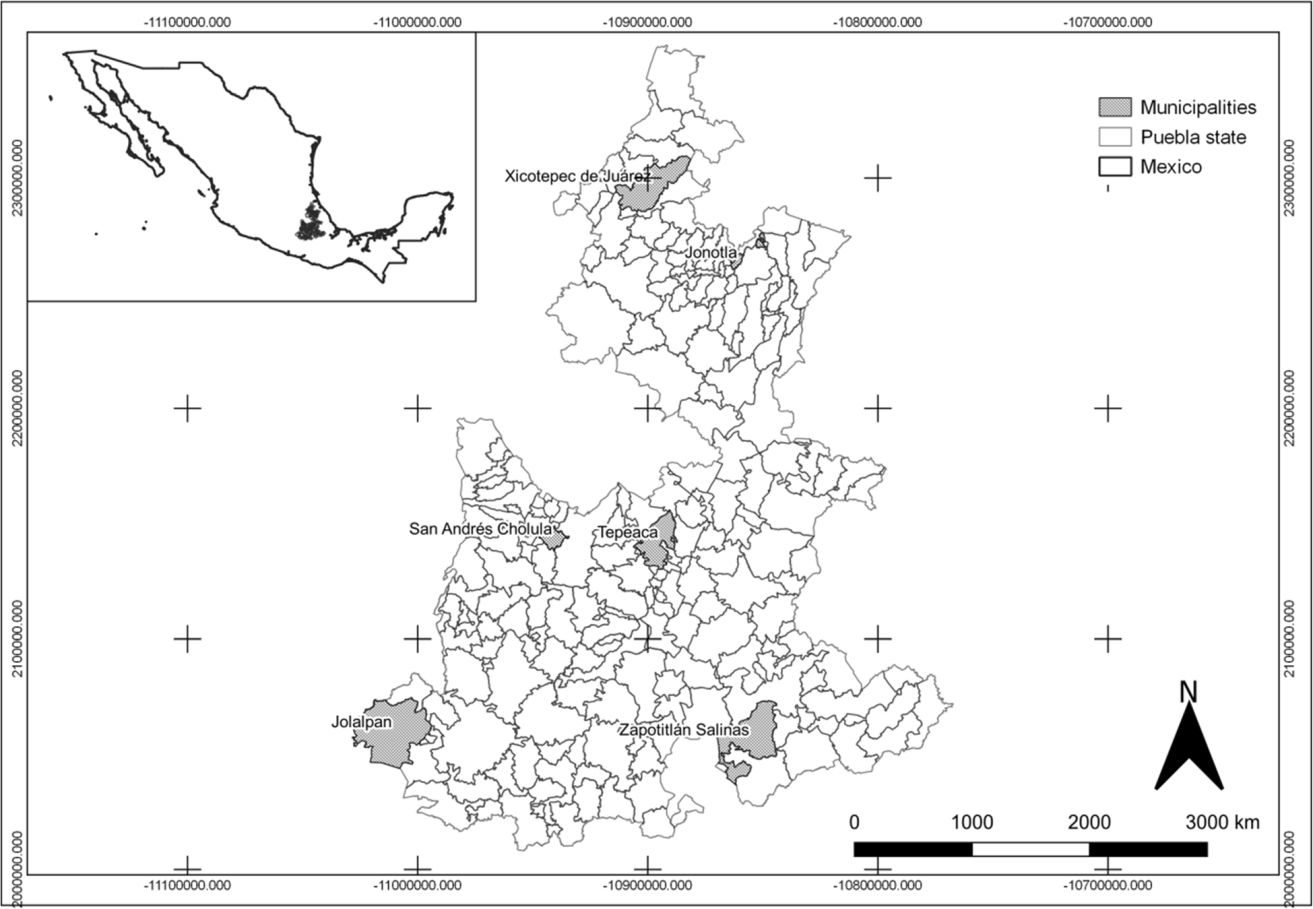
Geographic location of the municipalities in which the surveys were applied in the state of Puebla

The present study was conducted in six municipalities in the state of Puebla, in the central region of Mexico, located at 20° 50’ 24” N, 17° 51’ 39” S, 96° 43’ 29” E, and 99° 04’ 14” W [23]. Corresponding to two locations in the south, two in the center, and two in the north of the state (Fig. 1), these study locations were selected based on the cultural, environmental, and ecological differences among the regions of the state visited (north, center, and south) (Table 1).

**Table 1:** Characteristics of the communities where the surveys were carried out [24]

		Characteristics						
Regions	Municipalities	Coordinates	Altitude (masl)	Study site	Total population (inhabitants)	***T***	Precipitation (mm)	Climate
North	Jonotla	20° 00’ and 20° 10’ N, 97° 27’ and 97° 36’ W	100–1100	20	4,678	20–26 °C	2400–4100	Semi-hot humid
	Xicotepec de Juárez	20° 13’ and 20° 26’ N, 97° 45’ and 98° 02’ W	180–1700	108	71 454	16–26 °C	1900–2600	Semi-hot humid
Center	Tepeaca	18° 55’ and 19° 08’ N, 97° 48’ and 97° 58’ W	2080–2860	64	67,157	12–18 °C	600–900	Temperate subhumid
	San Andres Cholula	18° 59’ and 19° 04’ N, 98° 15’ and 98° 21’ W	2000–2180	23	80,118	14–18 °C.	800–1000	Temperate subhumid
South	Jolalpan	18° 10’ and 18° 26’ N, 98° 45’ and 99° 04’ W	700–1700	64	11,771	22–28 °C	800–1000	Hot subhumid
	Zapotitlán Salinas	18° 07’ and 18° 25’ N, 97° 24’ and 97° 41’ W	1200–2500	69	7774	14–22 °C	400–700	Semiarid temperate

### Sampling design

Directed sampling was carried out during the year 2015 at each study site and aimed to access those locations with the highest concentration of people (main squares, markets, city halls, etc.), in order to select the target population [25]. Target populations were divided by age ranges and sex, as follows: males and females between the ages of 12–32 (young people); males and females between the ages of 33–52 (adults); males and females older than 53 (older adults). We conducted an open-ended questionnaire, in which the respondent was asked to give three brief ideas (namely one expressed in a maximum of three words) that they related to the concepts discussed in each question (Supplementary Material 1). The sample comprised 60 questionnaires per community, with 20 questionnaires applied per age range (corresponding to ten males and ten females), thus obtaining a total of 360 questionnaires for all six communities sampled.

Before applying the questionnaire, the interviewer identified themselves as a student of the *Benemérita Universidad Autónoma de Puebla* (BUAP or Meritorious Autonomous University of Puebla) and informed the respondents of the purposes for which the data obtained would be used. Respondents were advised that the information would be used solely for research purposes, thus guaranteeing the anonymity and security of personal data under Mexican regulations, that participation was voluntary, and that they had the right not to answer any questions if they felt uncomfortable with them. In the case of respondents who were minors, the survey was conducted in the presence of parents or teachers. The questionnaire did not contain sensitive questions. These are questions that, under the Mexican law, affect the most intimate sphere of the respondent, or would elicit information whose improper use could lead to discrimination or put the respondent at risk, such as their sexual preferences or religion or whether they have been diagnosed with a specific illness. Only personal data that was strictly required by the present study was requested. Finally, the questionnaire was planned in such a way as to respect the respondent’s time and last no longer than 15 min, as well as reducing the use of paper as much as possible.

The questionnaire began by collecting categorical data, such as study site, age, sex, and the activity in which the respondent is engaged, and went on to ask the respondent to define an insect, of how many insects they were aware in the locality, and which insects they considered to be either beneficial or harmful. Finally, it contained questions related to SRs of insect-borne diseases, on such matters as prevention methods and the control of deleterious insects.

The answers obtained from the questionnaires went through a filtering process that consisted of eliminating synonyms and changing words from plural to singular, for example, words such as *chiquitos* (plural diminutive adjective for small), *animalitos* (plural diminutive adjective for animals), and *bonitos* (plural diminutive adjective for pretty). This filtering process was necessary to maintain the homogeneity of the expressions used without losing the meaning of the answers given [26].

### Data analysis

An SR can be considered as a structured and organized sociocognitive field. The data analysis undertaken in the present study aimed to identify the structure (central nucleus and peripheral system) and the level of organization of the corresponding SR, namely the degree to which the ideas that constitute the SR, are shared and based on consensus. The structure and the characteristics of the different groups sampled are described through both network analyses and correspondence analysis.

The organization of the SRs was analyzed by applying information indexes on the Hill numbers, thus enabling the establishment of the diversity of elements in an SR, which is understood as a function of the number of concepts within it and the homogeneity with which they are distributed. The number of concepts in the SR is indicated by N0, while the number of important concepts is indicated by N1, and the number of the most important and consensual concepts is indicated by N2. These numbers are calculated from the Shannon and Weaver index, which has a direct relationship with the information in a system and its complexity, and the Simpson index, which indicates the degree of which the information is heterogeneous. However, the interpretation of these indices is complicated, while the interpretation of Hill numbers is more intuitive [27]. It is assumed that, as an SR becomes increasingly complex in its organization, some concepts will appear more frequently than others, meaning that the N2 value will decrease. Table 2 shows how these indices are calculated [28].

**Table 2:** Formulas for the calculation of Hill diversity numbers

Index	Formula	Description
N0		Counts the total number of different opinions
***P***_***i***_	${P}_i=\frac{n_i}{N}\kern0.75em i=1,2,3\dots, C$	Where: *P =* proportional abundance of the *i*-belief: *n*_*i*_*=* frequency of the *i*-belief *N=* total number of T beliefs *P*_*i*_ is used to obtain diversity indices
Simpson’s diversity index (***λ***)	$\uplambda =\sum \limits_{i=1}^s{P}_i^2$	Indicates the dominance of some ideas over others, this enabling the degree of agreement about a represented object in a community to be ascertained
Shannon-Weaver diversity index (*H’*)	${H}^{\prime }=-\sum \limits_{i=1}^{S^{\ast }}\left({P}_{i\kern0.5em }\ln {P}_i\right)$	Where *S* is the known beliefs with proportions. Indicates the degree of complexity of the representation, wherein, as the number of opinions increases, the value of *H’*_max_ tends to be higher
***H***′_**max**_	*H*′_max_ = ln N0	Maximum diversity of opinions
N1	$\mathrm{N}1={e}^{H^{\prime }}$ *e* = 2.71828	Indicates the number of abundant beliefs in the SR. *e* is the base of natural logarithms and *H’* is Shannon’s diversity index
N2	N2 = 1/*λ*	Indicates the number of very abundant beliefs in the SR
Information index (*I*)	*I* = *H*′_max_ − *H*′	Indicates the amount of information in the SR
Organization index (*Q*)	$Q=1-\left(\frac{H^{\prime }}{H_{\mathrm{max}}^{\prime }}\right)$	Indicates the degree to which the information is organized

### Structure: network analysis

Network analyses based on graph theory enable the structure of an SR to be ascertained by determining the interaction between words [28]. A graph or sociogram is composed of a group of nodes (points that can represent actors, words, or ideas) and a group of lines (relationships or links), with the connection between these line and node components helping the visualization of the frequency, connectedness (node size), and relationship (line width) for each of the ideas [29]. In order to obtain the network for the analysis, the weighted matrix was processed in UCINET 6.5 [30], and it was drawn using Netdraw 2.1 [31]. After constructing the networks, the following measures were calculated:

#### Centralization measure

According to Guimelli [32], the elements of the central nucleus of the SR are characterized by a high incidence of association with other elements. This measure indicates the condition under which ideas play a strictly central role, wherein the surrounding nodes need to pass through the central node to connect, as it is closely connected to the network, giving a centralization degree of 100%. On the other hand, centralization degrees with low values indicate the absence of clearly central ideas.

#### Density measure

This measures the degree of intensity of the connectivity of the network. A network with high connectivity indicates that its ideas interact with each other and that it contains many pathways connecting one idea to another [32, 33]. A dense SR is assumed to be poorly organized and unstable.

### Group characteristics: correspondence analysis (CA)

Correspondence analysis can be considered as a method of exploratory analysis that can describe the relationship between different qualitative variables, in this case, the ideas expressed in response to the verbal association tasks in the questionnaire. This form of analysis is based on a contingency table which breaks down the chi-squared distance into orthogonal factors. Due to its exploratory character, CA can be performed even when the cell count in the contingency table is less than five. However, responses with a low frequency tend to be far from the center of the graph, giving the impression that they are of high value as indicators for differentiating between groups, for which reason, it is advisable to eliminate the answers with a frequency of less than 2% [34]. Hill numbers were used as a criterion to eliminate the infrequent ideas and solely analyze the important ideas (N1), while the replies that were not considered were included in the category *Other*.

Correspondence analysis enables a link to be found between different components of the SRs and the relationships among them, as well as the social insertions of the individuals within the groups sampled [35]. Relationships can be observed via either a Cartesian diagram or a perceptual map, in which clusters (concentrations of points) that describe a particular pattern can be formed. When the points are located at the center of the axis, this indicates a strong interconnection between the variables, making it difficult to measure their individual effects on the response variable (variable of interest) [36]. Correspondence analysis also enabled the identification of differences among groups sampled, while a chi-squared test was performed to determine which study group (independent variables) presented different answers.

## Results

### Indices of SRs, attitudes, and practices that people have regarding insects

The analysis of the data obtained from all six communities of interest in the state of Puebla shows that their members present a great diversity of knowledge about insects or animals that they consider as such (94), identifying 43 animal species living in their homes, 59 living exterior to their homes, and 37 causing diseases. The respondents referred to the same number of insects that they liked and dislike (55). In general, this information is poorly organized, as the very important species (N2) account for between a quarter and a third of the above.

The respondents’ definitions of what an insect is, the diseases that they cause, and the actions people take after being bitten, are much more organized and based on consensus indicating between five and ten important ideas (Table 3).

**Table 3:** Information indices of SRs, attitudes, and practices that people have regarding insects

Concept	Hill diversity numbers		
	N0	N1	N2
Definition of an insect	69	20	10
Information sources	16	7	5
Diversity of insects	94	37	26
Insects you like	55	25	15
Why do you like them?	67	23	12
Insects you dislike	55	23	15
Why do you dislike them?	61	17	9
Insects inside the house	43	15	11
Insects outside the house	59	32	24
Prevention methods	55	23	16
Actions after a bite	45	9	5
Insects that cause diseases	37	15	10
Known diseases	45	13	6

### Representation scope component: network analysis

Network analyses were carried out considering two categories: (a) knowledge, perception, and relationship and (b) attitudes and practices. A summary with a detailed explanation of each category is given below, indicating the concept represented by each question, the ideas comprising the central nucleus and the main relationships in the network, the peripheral system, and the density and centralization degrees of the networks (Tables 4 and 5). For example, for *Definition of an insect*, three ideas (*little animal*, *small*, *and flying*) comprising the central nucleus of the network were identified, while the words *bites*, *bug*, *disease*, *little legs*, *creeping*, *harmful*, and *pest* comprise the peripheral system. In the case of the density and centralization percentages, it is observed that there are no clear central ideas in the network, which indicates that the ideas interact with each other and the possibility of pathways connecting one idea to another.

**Table 4:** Summary of network analysis indicating the central nucleus, peripheral system, density, and centralization percentages (knowledge, perception, and relationship)

Concept	Central nucleus	Peripheral system	Density	Centralization
Definition of an insect	Little animal, small, and flying	Bites, bug, disease, little legs, creeping, harmful, and pest	49.2%	23.6%
Information sources	Subscription television, children’s movies, and biology books	Newscasts and movies in general	38.8%	30.1%
Insects you like	Butterfly, grasshopper, and bee	Cricket, ant, beetle, spider, larva food, true bug food, dragonfly, ladybug, scorpion, wasp, caterpillar, and worm	30.8%	27.8%
Insects you dislike	Mosquito, cockroach, and fly	Scorpion, spider, ant, mosquito, bee, wasp, flea, worm, and centipede	79.5%	20.8%
Insects that cause diseases	Mosquito, scorpion, and fly	Cockroach, spider, and flying bug	66.8%	45.2%
VBDs	Dengue, fever, hives, malaria, and chikungunya	Intestinal infection and poisoning	34.8%	40.7%

**Table 5:** Summary of network analysis indicating the central nucleus, peripheral system, density, and centralization percentages (attitudes and practices)

Concept	Central nucleus	Peripheral system	Density	Centralization
Prevention methods	Repellent, cleaning, and fumigating	Killing the animal, insecticide, mosquito coil, covering up, mosquito net, not bothering, precaution, closing doors, scaring them away, Raid, mosquito screen, powder poison, and home remedies	43.6%	31.5%
Actions after bites	Home remedies, scratching, and doctor	Ointment and VapoRub	79.6%	38%

### Group characteristics

We found that differences in the social representations are identified in the variables *region* and *age*, with significant values obtained for twelve out of the thirteen questions on the questionnaire (*p* < 0.05). The variable *sex* did not present sufficient influence to produce a difference in the SR, as only four out of the thirteen questions (*Information sources*, *Insects you dislike*, *Why do you dislike them*, and *Actions after a bite*) presented significant values (*p* < 0.05). In terms of the question that asked respondents to identify insects that cause diseases, significant differences were solely observed for the variable *region*, while, similarly, significant differences were solely observed for *definition of an insect* with the variable *age*.

### Correspondence analysis (CA): SR of insects in the state of Puebla

**Fig. 2:**
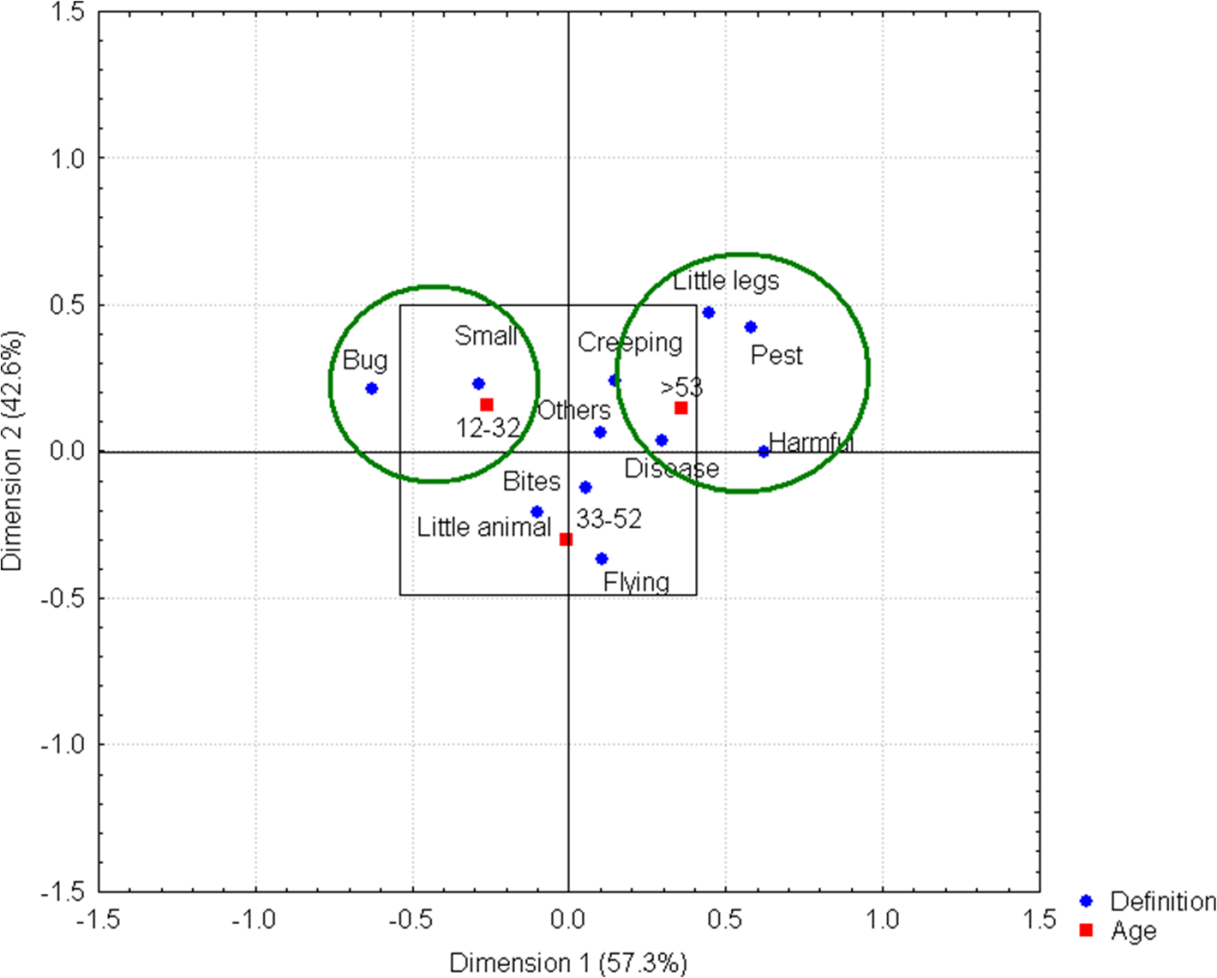
Definition of an insect by age range. The two connections between the ideas and the 12–32 age range and the respondents over the age of 53 years are shown within the ellipses. A cluster of words can be observed at the center of the graph, thus significantly representing respondents’ opinions, given that no relationship with any age range was found

Describing or defining an insect, taking its size into account, can be somewhat difficult for some people. However, the answers given by the people interviewed in the present study refer to the morphological characteristics of these organisms or to the problems they can cause, either in the form of diseases in humans and animals or as pests of crops. Respondents in the 12–32 age range associate this group of organisms with the words *bug* and *small*, while those older than 53 years associate insects with words *little legs*, *pest*, and *harmful*. The cluster of words at the center of the axis indicates that these ideas represent a high consensus value for the respondents, namely that there is no strong relationship with any of the age ranges (Fig. 2).

**Fig. 3:**
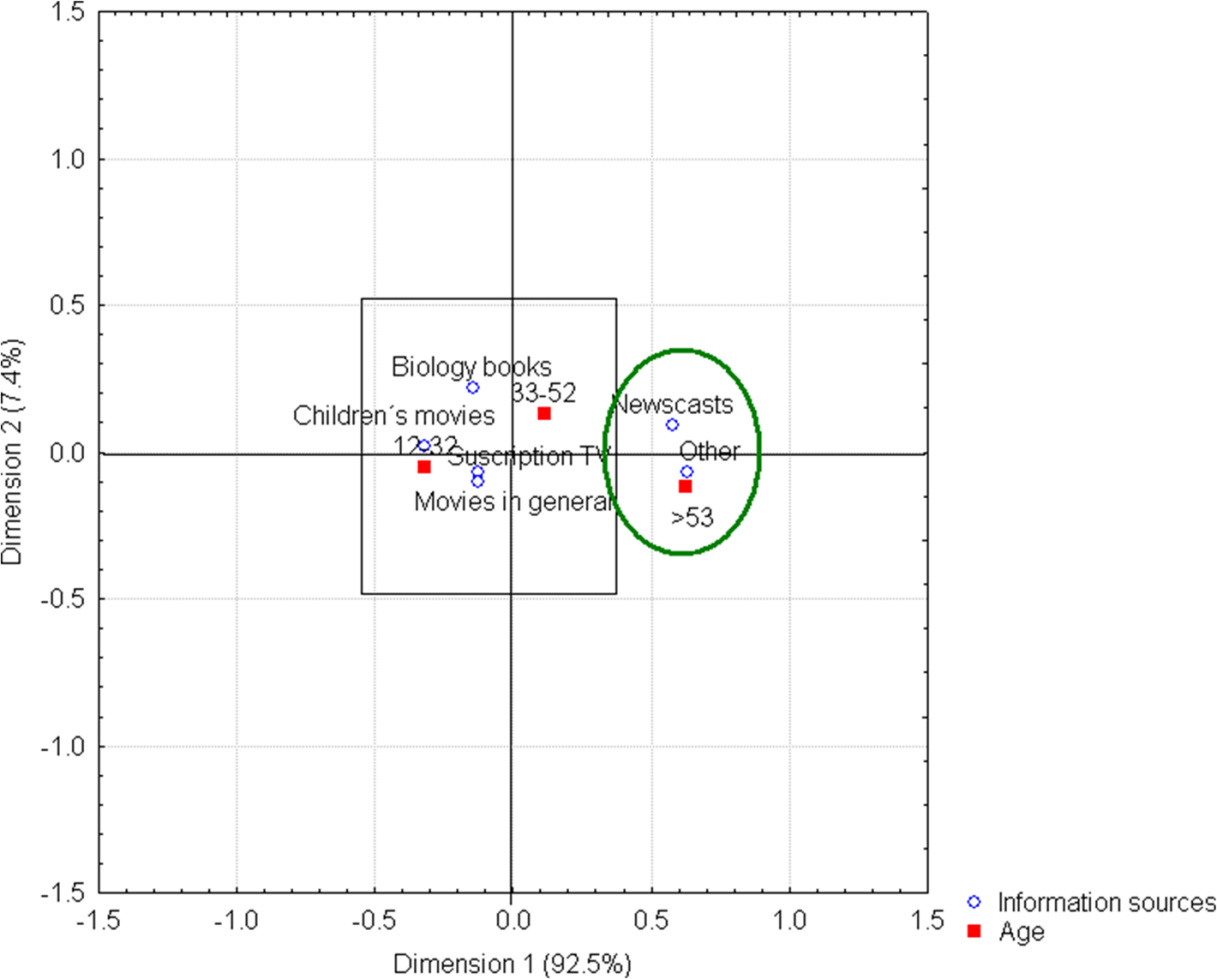
Information sources by age range. It is possible to observe the main link between television (newscasts), as a source of knowledge about insects, and people older than 53 years

Television appears to be the most viable option for *information sources*, as this medium may be reliable and accessible at the desired time and place and is, therefore, the main option for receiving knowledge and constructing an SR of insects. For this reason, *Newscasts* were the main information source for people older than 53 years, which could be due to the fact that most of those in this age range, both male and female, are able to spend most of their time at home (Fig. 3).

**Fig. 4:**
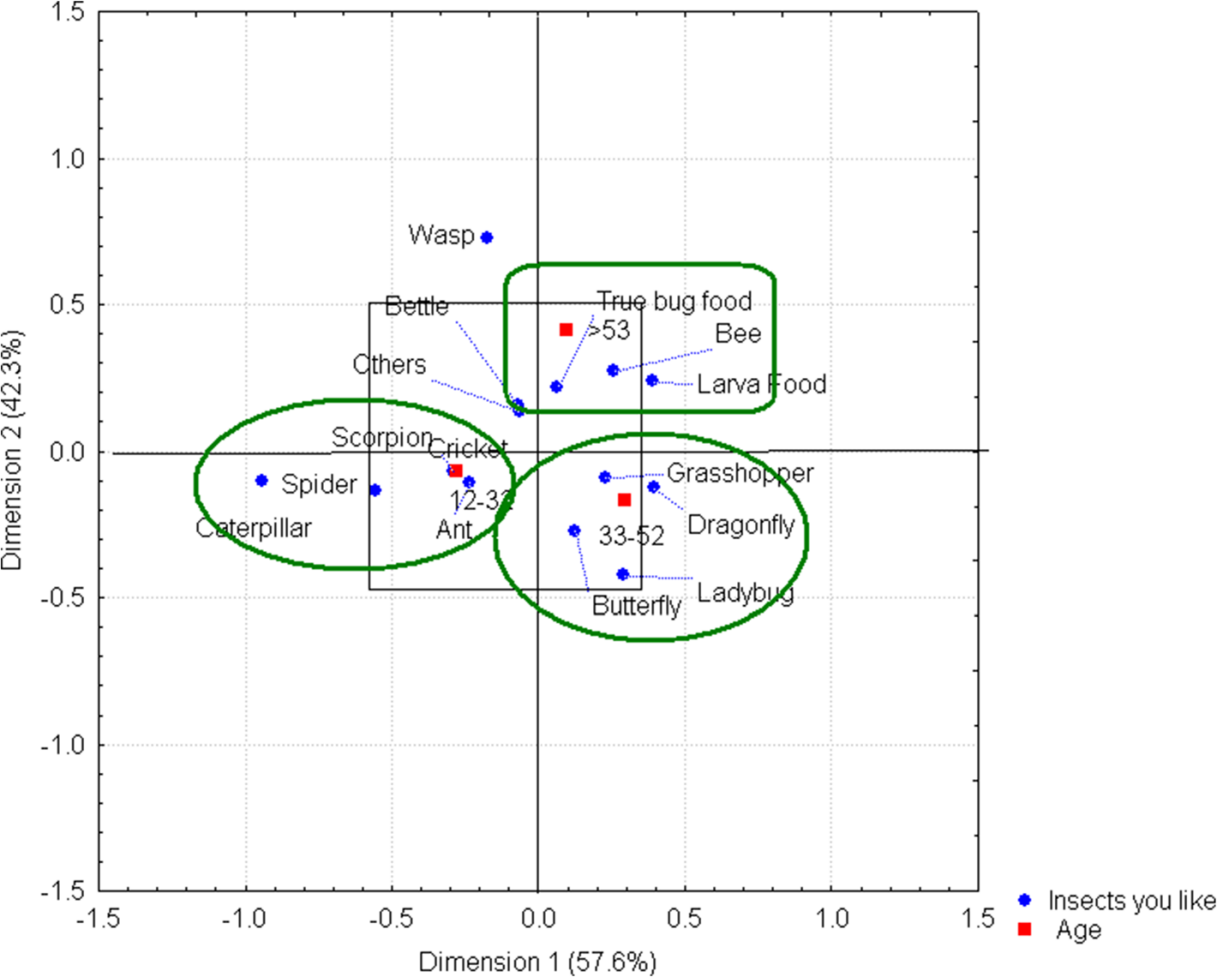
Insects that people like by age range. Three primary links can be observed within the ellipses. People in the 12–32 and 33–52 age ranges are attracted to insects because of their diversity in shapes and colors. On the other hand, people older than 53 years are more interested in the economic and nutritional benefits they obtain from selling and consuming these organisms

The types of insects that people like differ among age ranges and are mainly determined by the benefits that people obtain from them. Some insects are viewed positively because of the diverse shapes and colors, with the 12–32 age range preferring insects such as caterpillars, crickets, ants, spiders, and scorpions (last two are arachnids and not insects). Dragonflies, grasshoppers, butterflies, and ladybugs are associated with people in the 33–52 age range. Other organisms such as true bugs, butterfly larvae, and bees are of interest to people older than 53 years, as residents of the communities sampled obtain economic benefits from producing and selling honey and obtain nutritional benefits from the consumption of certain insect species as part of their diet (Fig. 4).

**Fig. 5:**
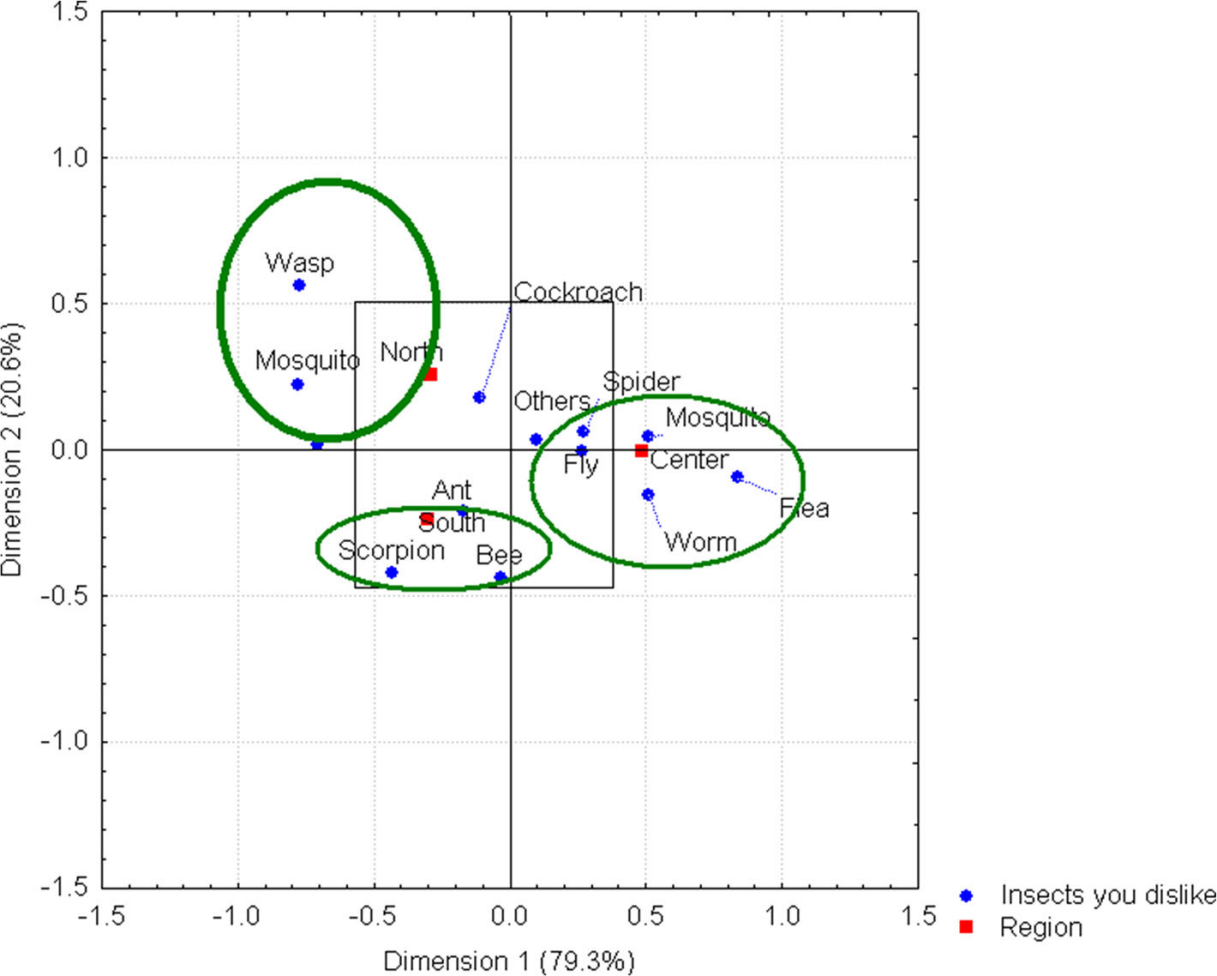
Disliked insects by region. Two relationships, each indicating dislike for a distinct pair of insects, can be observed for the northern and southern regions of the state. In the case of the central region of the state, a link indicating dislike for four insects can be observed

Even though some insects are attractive to some respondents, others cause entomophobia, a very common phobia, which was identified in the three regions sampled. In the northern region, a dislike for insects such as wasps and mosquitoes was expressed, while respondents in the central region of the state expressed a dislike for flies, mosquitoes, worms, and fleas. Finally, respondents in the southern region expressed a dislike of scorpions and bees (Fig. 5).

**Fig. 6:**
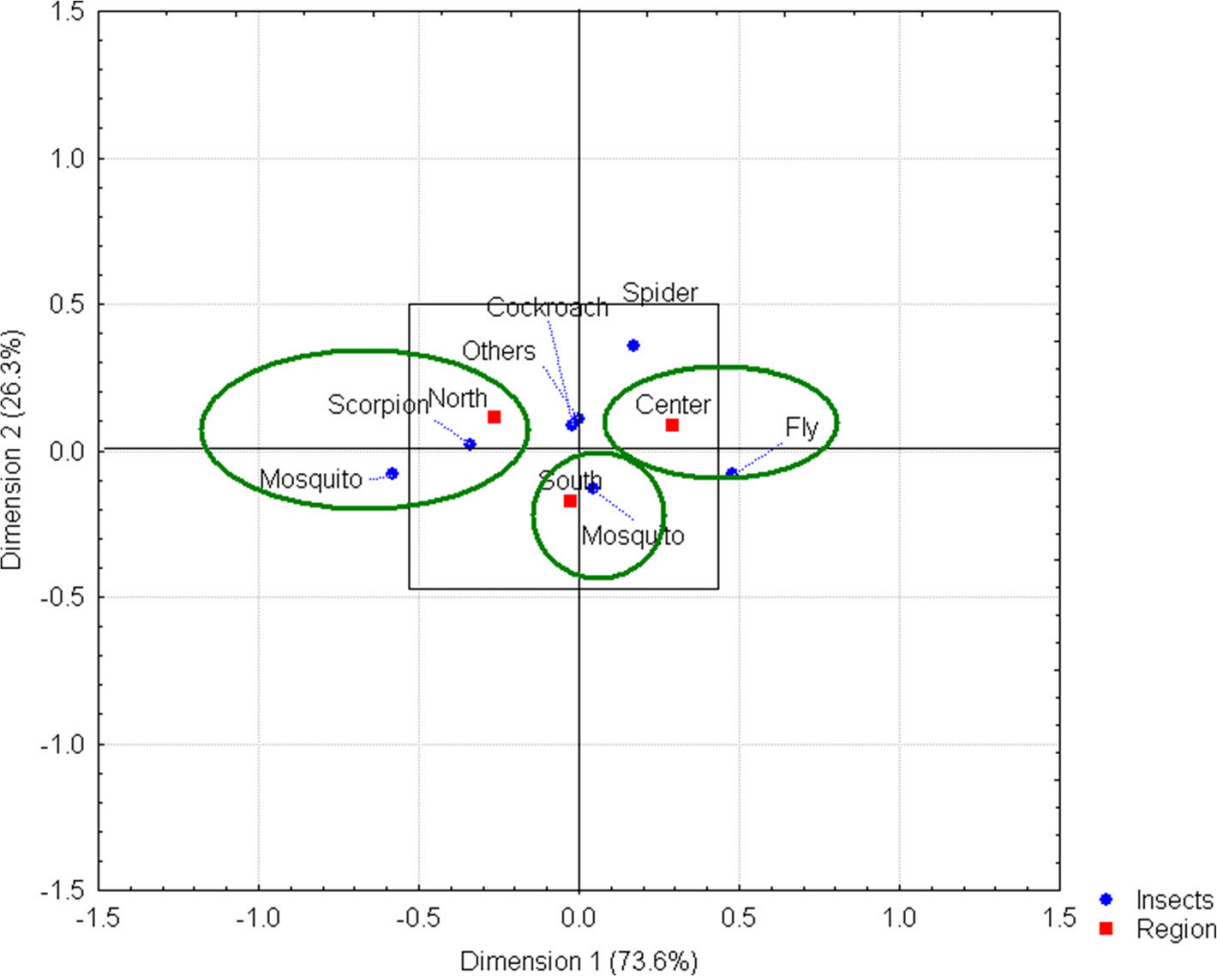
Insects that cause diseases by region. The relationship between the fly and the central region of the state is shown, as well as the relationship between the mosquito and the northern region. However, the strongest link is between the mosquito and the southern region, as indicated by a highly significant value, and is reflected in its location at the center of the graph

By region, specific knowledge on insects that transmit diseases is observed, which can be explained by the geographical distribution of the organisms and the frequency with which people are exposed to them both inside and outside their homes. Two insects were mentioned as the main vectors of transmission, with the mosquito and the scorpion identified by respondents in the northern region of the state, while those in the central region identified such a relationship with the fly. However, it should be noted that the link between the southern region and the mosquito as a vector as the transmission is a highly significant value, as reflected by its position at the center of the axis of the graph (Fig. 6).

**Fig. 7:**
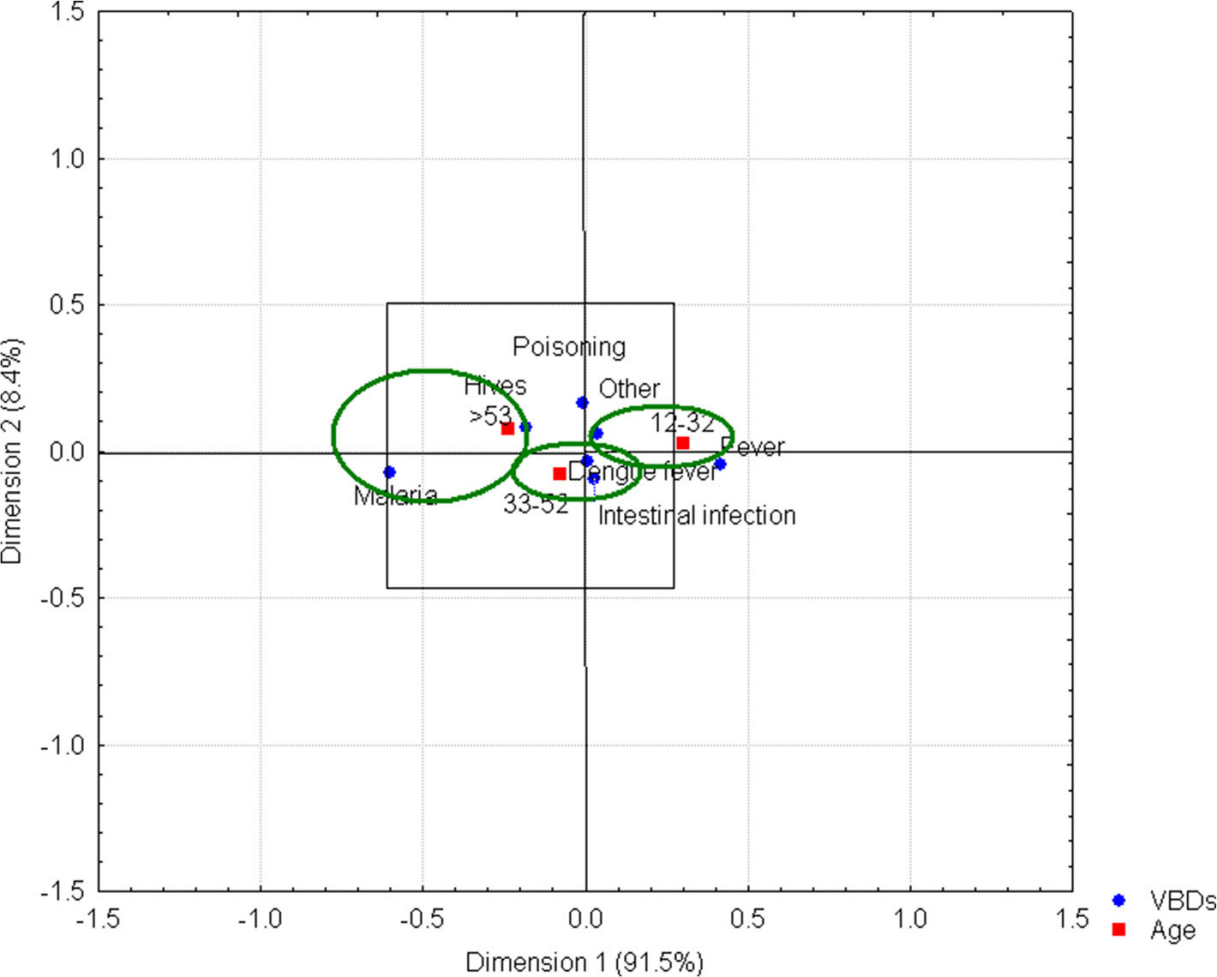
VBDs by age range. Ellipses indicate the relationship between malaria and people older than 53 years, as well as the link between fever and people in the 12–32 age range. However, dengue fever disease is located at the center of the graph, indicating a strong interconnection for the population sampled

Knowledge about VBDs by age range is somewhat confusing for people as both symptoms of a disease and the secondary effects of a bite are observable, as are poisoning and intestinal infections, which are not always directly caused by insects. The relationship observed between people older than 53 years and malaria may be due to their exposure to a high number of infections when they were young. Fever, the main symptom of dengue fever, was associated with people in the 12–32 age range, while dengue fever, hives, intestinal infection, poisoning, and other problems are located very close to the central axis of the graph because they are the most common diseases (Fig. 7).

### Correspondence analysis: attitudes and practices regarding insects in the state of Puebla

Although the respondents were aware of different cultural and physical control measures, such as home cleaning, the use of mosquito nets and screens, covering up the skin, and the general application of caution to prevent insect bites, they still indicate chemical control measures, particularly insect repellent, as the most commonly used method. Therefore, clear correspondence relationships in the graph cannot be observed, as all answers and variables are located close to the central axis, thus indicating that chemical prevention methods are of great importance for respondents (Supplementary Material 2 and 3).

Actions taken to treat insect bites are similar by both region and age range. The corresponding cluster of words (*home remedies*, *scratching*, *seeing a doctor*, *applying ointments*, and *VapoRub*) and variables (age ranges and regions) are located very close to the center of the axis, showing a high level of consensus for this group of answers in the state of Puebla. Traditional medicine (*home remedies*) and self-medication are the measures that respondents apply first (Supplementary Material 4 and 5).

## Discussion

When exploring the SRs, attitudes, and practices in the state of Puebla, we observed that there is no clear definition of the word *insect*, as the respondents associated all very small flying animals with this word. They mainly mention morphological characteristics that can be seen with the naked eye or that they have seen in movies or TV shows, as television was the most significant source of information for the communities sampled, a finding that coincides with that reported by Boratne et al. [37].

As the health sector does not provide enough information regarding the prevention and control of VBDs, respondents in the communities do not consider this sector as an alternative source of information, a finding that coincides with that reported by Griffith et al. [12] in Panama and by Rosecrans et al. [15] in the state of Yucatan, Mexico.

The distribution of information on VBDs in Puebla has not been sufficiently effective and has created limitations that result in the respondents exhibiting knowledge of only one group of organisms and the diseases they transmit [38, 39]. This leads to the neglect of and failure to attend to other diseases such as leishmaniasis, Chagas disease, and even rickettsiosis. This can be seen with vector insects that are found in the southeast of the state, such as the kissing bug (*Meccus pallidipennis*) which transmits the causal agent of Chagas disease [40], and different mosquitoes and sandfly species which transmit viral diseases and leishmaniasis, respectively [38, 39, 41].

The foregoing notwithstanding, respondents mainly identify mosquitoes as the main vector transmission [9, 10] and dengue fever as the main disease, coinciding with that reported by Chuc et al. [13] and Torres et al. [14] in the states of Morelos and Chiapas. However, these studies also report that malaria and chikungunya are also mentioned as disease caused by insects in these states, as well as some secondary effects, such as hives, fever, intestinal infections, and poisoning, despite the fact that they are not necessarily transmitted by insects. These limitations, which create confusion in terms of knowledge about insects and VBDs, can be overcome by means of more efficient community education campaigns about the elimination and control of these diseases and which aim to maintain and expand a culture of prevention, mainly in places with a latent risk of an outbreak of one of these diseases [10, 11].

In order to determine the relationship between people in the communities sampled and insects, respondents were asked which insects they liked and which they disliked. The butterfly, the bee, and the grasshopper were described as insects they liked, which could be due to beliefs about these animals, their bright colors, the sounds they produce, or their beneficial properties.

The symbolism of butterflies varies among study sites and individuals, with their representation associated with different lifestyles, cultures, religions, and beliefs [42]. For many people in the state of Puebla, lepidopteran species are associated with superstitions with different meanings. For example, moths are considered to be bad omens and are associated with bad luck and death, white butterflies are associated with good luck and presage changes in the weather, and other butterfly species are associated with economic gain or loss. Some species attract attention because of their diverse colors and their process of metamorphosis, while some are even associated with palliative care [42].

Entomophagy (the human consumption of insects) is a significant practice in the state of Puebla. Edible insects, such as *chicatana* ants (*Atta mexicana*), grasshoppers (*Sphenarium purpurascens*), *chinicuiles* (moth larvae) (*Comadia redtenbacheri*), and *jumiles* (small stink bugs) (Pentatomidae) can be considered as an alternative food source in the region because of their high nutrient content. They could, therefore, be useful in counteracting malnutrition in many regions of the country [43, 44].

As apiculture is another significant activity in the communities sampled, this may explain why respondents indicated a liking for bees given the personal or economic benefits obtained from the sale of honey and products derived from it, both nationally and internationally [45].

The results obtained by the present study showed that entomophobia is associated with the density, size, shape, smell, and colors of the specific insect, as well as with the places where it lives. This fear also stems from the bad reputation attached to them as a result of them being depicted with superhuman size or considered as pests or bad omens or, simply, their appearance [6, 46]. Furthermore, the feeling of repulsion or rejection exhibited towards insects such as cockroaches, mosquitoes, and flies have a historical, ethnic, or cultural explanation and is a natural response, given that these organisms are directly associated with a lack of hygiene, food contamination, and the spread of diseases causing vomiting, diarrhea, and nausea. All the foregoing contextual explanations and beliefs were expressed in the present study.

With regard to attitudes and practices relating to the control of insects, chemical control measures (insect repellent) are the main prevention method. This finding is similar to that reported by Rosecrans et al. [15] and can be explained by the fact that the direct application of a spray or ointment on the skin is an easy and quick way to preventing contact with insects. However, this method does not eliminate insects and only repels them for periods ranging from 10 min to 5 h, depending on the chemical composition [47]. For example, some repellents contain sunscreen and N,N-Diethyl-meta-toluamide and N,N-Dimethylbenzamide (DEET), and are not recommended by the United States Centers for Disease Control and Prevention (CDC) because of the limited efficacy of both components [48].

Mexico’s Ministry of Health [49] promotes cultural insect control measures, such as personal hygiene, wearing clothes that cover the skin, cleaning, housekeeping, and the removal of breeding sites. While access to potable water and sanitation are also important factors for the control and elimination of diseases, such improvements within the home are costly and families are not always able to afford them, meaning that they often prefer to continue with lifelong household practices [50].

In terms of the actions taken in response to an insect bite, respondents report that they usually resort to traditional medicine and home remedies (applying garlic, lime, aloe vera, herbal tinctures with alcohol, etc.), as well as to self-medication. These methods are applied because they are found to be more reliable, given that they correspond to acquired knowledge based on trust and beliefs and practices that have been passed down from generation to generation. Their use also persists because of the inefficiency of local health services and the common lack of economic resources required to access private healthcare [50, 51]. It should be noted that home remedies are effective in alleviating some of the discomforts caused by insect bites, such as inflammation, irritation, and even infections; however, these remedies cannot be used as clinical treatments to cure VBDs such as dengue fever, chikungunya, Zika, or Chagas disease.

## Conclusions

The present study found that after comparing the different SRs of insects and VBDs in the regions of interest, television plays an essential role in providing the information people require. However, this media source does not provide information of sufficient quality on the control and prevention of VBDs to enable communities to know how to protect themselves and their communities. Dengue fever and mosquitoes are the disease and insects, respectively, that are most commonly known among communities sampled. We identify the need for both guidance and the constant prevision of information about other endemic VBDs considered to be neglected in the public health sector, such as Chagas disease and leishmaniasis.

Entomophobia is the main cause of the aversion demonstrated to these organisms by the respondents of the questionnaires applied by the present study. The insect repellents used to prevent VBD transmission in the communities sampled are not efficient, while methods with better results and which help to eliminate the vector, such as cultural control measures (cleaning and the removal of breeding sites), are not adopted. Moreover, the practice of traditional medicine and home remedies is still applied in these communities, as they help to control the secondary effects of an insect bite; however, these practices are not recognized by health professionals as clinical treatments for these diseases. Finally, research such as the present study can be highly useful for the promotion of training and education programs focused on creating strategies for the prevention and control of VBDs, especially those (Chagas disease and leishmaniasis) that are endemic but neglected by general population.

## Supplementary Information

**Additional file 1.** Questionnaire and graphs on prevention by age ranges and regions and actions by age ranges and regions.

## Data Availability

The data generated during the present study are available from the corresponding author on reasonable request.

## Abbreviations

VBD: Vector-borne diseases
SR: Social representation
