# Supplementary material for: Knowledge, attitudes, and practices regarding vector-borne diseases in central Mexico
Source: J Ethnobiol Ethnomed. 2021 Jul 21;17:45. doi: 10.1186/s13002-021-00471-y (PMC8296709; doi:10.1186/s13002-021-00471-y)
Supplement: Supplementary file 1 — Additional file 1. Questionnaire and graphs on prevention by age ranges and regions and actions by age ranges and regions. [file 13002_2021_471_MOESM1_ESM.docx]

**Supplementary material**

1. Questionnaire

Insects of public health importance in the state of Puebla

The purpose of the present questionnaire is to know the interpretation that residents of 6 municipalities of the state of Puebla have about insects. Please provide brief answers to the following questions. It is important that you answer the first thing that comes to your mind without thinking too much about the answer.

Age ____

Sex: Male 🞏 Female 🞏

How many years have you lived in this locality?___________

The main activity that you do

___________________________________

1.-For me, an insect is:

______________, _______________ and _______________.

2.-Mention 3 television shows, movies or books that portray insects.

_______________, _______________ and _______________.

3.- Mention 10 insects that live in your community

_______________, _______________, _______________, _______________, _______________, _______________, _______________, _______________, _______________, _______________.

4.-Mention 3 insects that you like

_______________, _______________ and _______________.

5.- Mention 3 reasons why you like them

_______________, _______________ and _______________.

6.- Mention 3 insects you dislike

_______________, _______________ and _______________.

7.- Mention 3 reasons why you dislike them

_______________, _______________ and _______________.

8.-Mention 3 insects that you see inside your house

_______________, _______________ and _______________.

9.- Mention 3 insects that you see outside your house

_______________, _______________ and _______________.

10.- Mention 3 ways to prevent insect bites

_______________, _______________ and ________________.

11.-Mention 3 things you do when an insect bites you

_______________, _______________ and _______________.

12.- Mention 3 insects that cause diseases

_______________, _______________ and _______________.

13.- Mention the diseases that these insects cause

_______________, _______________ and _______________.

**Supplementary material**


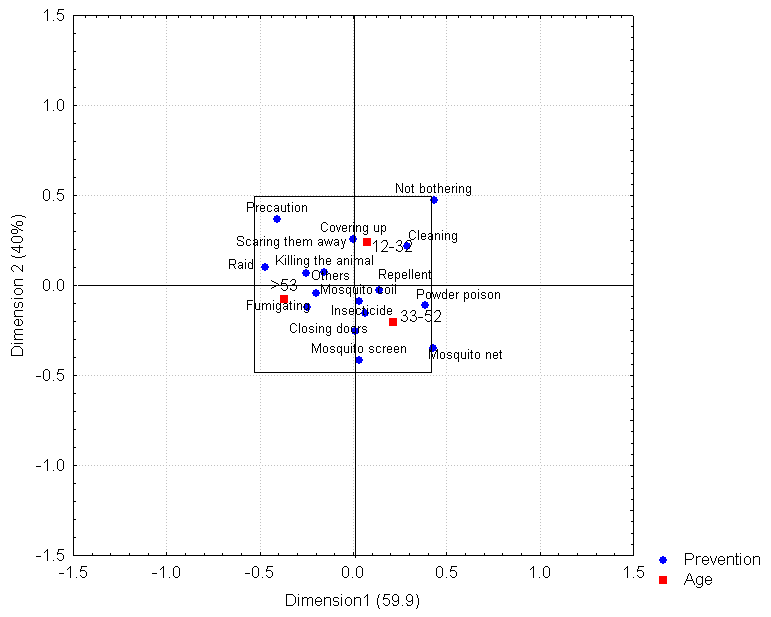


1. **Preventions by age ranges**. The evocations “repellent” and “mosquito coil” (chemical control) can be observed at the center of the graph. These answers are strongly related to the population. Age groups are also close to the center of the graph; thus, it is difficult to measure the specific effects of the variable.


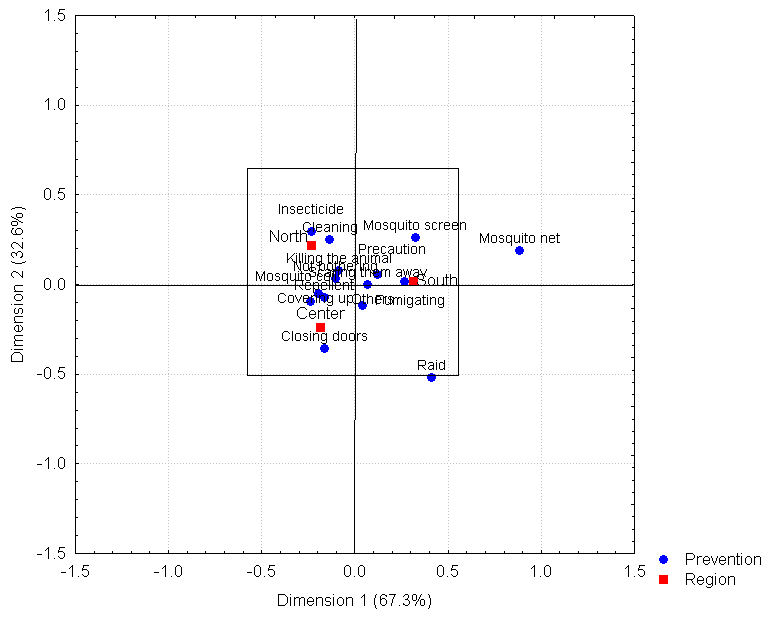


2. **Preventions by regions**. A cluster of evocations is located at the center of the graph. “Repellent” and “mosquito coil” (chemical control) are the most representative answers in the three study zones. The graph also shows no relationship between evocations and regions, since the variables are strongly interconnected, which makes determining their individual effects difficult.


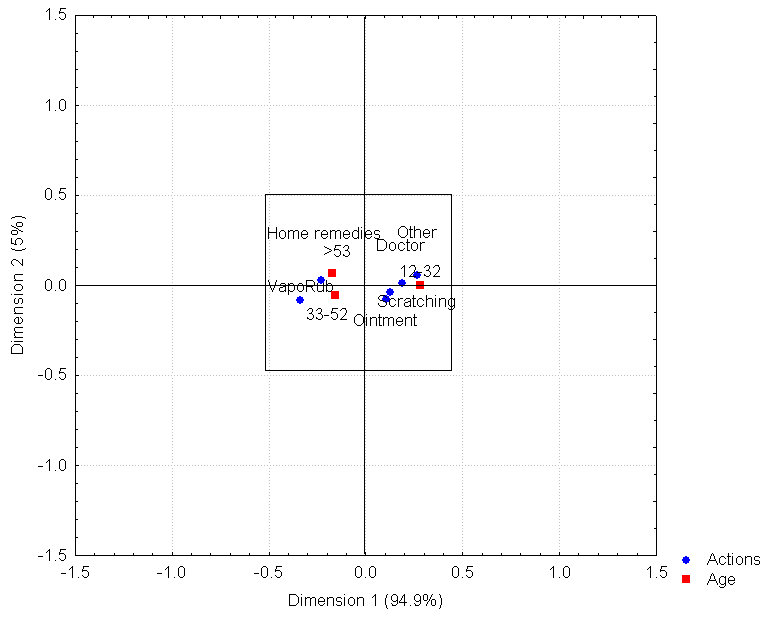


3. **Actions by age range.** There are no interconnections since the group of evocations and the age ranges are close to the center of the graph. Therefore, this group of answers shows a high significant value for people, independently of age range. The practice of traditional medicine (home remedies), as well as self-medication, are the activities that people resort to first.


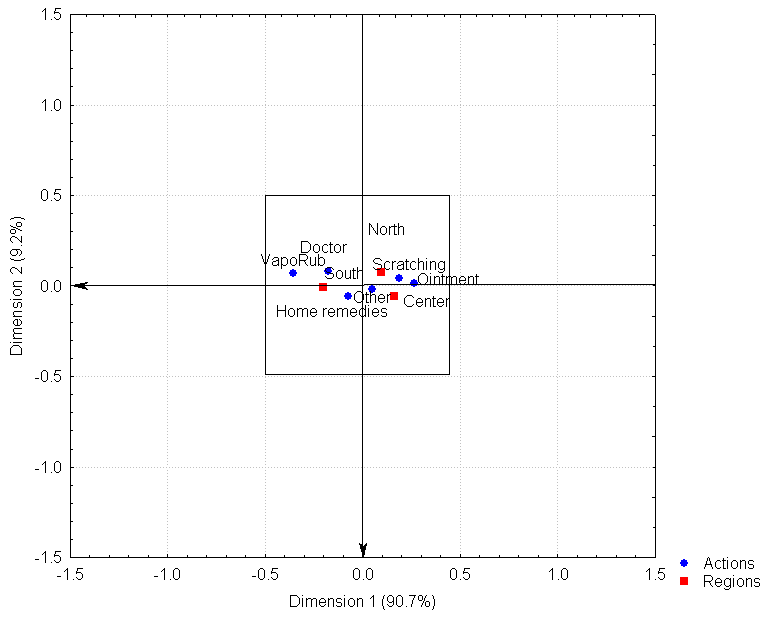


4. **Actions by regions.** The three regions (north, center, and south) and the evocations are clustered at the center of the graph. Thus, there is no correspondence relationship between answers and regions. Therefore, this cluster of answers has a high significant value for the three regions. Home remedies and self-medication are the most common actions in the communities.
